# Preliminary Design and Development of a Mechanical Ventilator Using Industrial Automation Components for Rapid Deployment During the COVID-19 Pandemic

**DOI:** 10.7759/cureus.20386

**Published:** 2021-12-13

**Authors:** Benjamin DeBoer, Ahmad Barari, Mika Nonoyama, Adam Dubrowski, Marco Zaccagnini, Ali Hosseini

**Affiliations:** 1 Faculty of Engineering and Applied Science, Ontario Tech University, Oshawa, CAN; 2 Faculty of Health Sciences, Ontario Tech University, Oshawa, CAN; 3 Respiratory Therapy, McGill University, Montreal, CAN

**Keywords:** real time operation, rapid development, industrial automation, mechanical ventilator, covid 19

## Abstract

The novel coronavirus disease 2019 (COVID-19) created a shortage of mechanical ventilators in the healthcare sector, resulting in rationed distribution, ethical dilemmas, and high mortalities. This technical report outlines the design and product outcome of a mechanical ventilator based on readily available off-the-shelf components, minimizing the dependence on manufacturing facilities. The ventilator was designed to operate in both hospitals and remote locations, having the ability to operate off various gas pressures and low voltage supplies. Due to the COVID-19 restrictions, the challenges of developing a device in an online setting with minimal manufacturing assistance were explored. Within a 10-day period, the team designed, prototyped, and conducted preliminary feasibility testing on the mechanical ventilator. The proposed design was not intended to replace, or be used as a medically approved ventilator, but demonstrates the ability to exploit off-the-shelf components to enable fast development and assembly.

## Introduction

There have been over 185 million confirmed cases of the 2019 coronavirus disease (COVID-19) worldwide and over four million deaths (data as of July 2021 [1]). During the “first wave” of the COVID-19 pandemic, approximately 5% to 10% of COVID-19 infected patients required admission to the intensive care unit (ICU) and required support from a mechanical ventilator, a form of life support machine that supports the work of breathing from patients unable to breathe on their own [2]. This large and rapid influx of patients requiring intensive resources caused a major strain on health care systems globally. Some health care systems (e.g., Italy and New York) struggled to meet the immediate and substantial increased demand for mechanical ventilators, creating a shortage worldwide. As a result, some countries reported that health care professionals had to triage and ration mechanical ventilators for patients with COVID-19, resulting in high mortalities and difficult ethical dilemmas [3-5]. As cases continue to rise and multiply globally, it is vital to have intensive and creative solutions to cope with shortages in life-supporting equipment and supplies.

Across Canada, there are approximately 5000 available mechanical ventilators, with over 2000 in Ontario (the largest province) [6]. In 2019, the ventilator stockpile in Ontario was 209 across 14 hospitals [7]. Considering the rising number of COVID-19 cases, the emergence of COVID-19 variant strains, and the possibility of more patients requiring intensive care and mechanical ventilation support, the availability of mechanical ventilators may be in short supply. One possible solution to increase the availability of mechanical ventilators is to construct machines that are simple, low-cost, and easy to operate and manufacture. These can be mass-produced to address current and future equipment shortages, mass casualty scenarios or pandemics. Since the COVID-19 pandemic, there has been a plethora of rapidly developed and low-cost mechanical ventilators designed in response to potential shortages [8-10], many of them open-source [11]. These open-source designs provide stakeholders (e.g., engineers, health care professionals) with different options of mechanical ventilator designs, materials, and/or processes that best fit with their environment and available resources and provide a template for future researchers and engineers to improve on mechanical designs.

The purpose of this technical report is to describe the details of the design and development of our prototype mechanical ventilator, and the team's process to complete this project.

## Technical report

The technical report is separated into four sections [12]: the context, the input, the design process, and the end-product/outcomes. The context outlines the features and potential scenarios for the mechanical ventilator, followed by the input of resources (infrastructure, human resources, components, materials) required to design and construct a prototype. The design process presents the high-level methodology behind building the ventilator, with the initial results and feasibility shown in the end-product/outcome section.

Context

The Montreal General Hospital Foundation and the Research Institute of the McGill University Health Centre attempted to address Canada's potential shortage by creating an "open-challenge" for engineers and front-line clinicians to design a "low cost, simple, easy to use and easy to build ventilator that can serve COVID-19 patients" within 10 days. The medical compliance requirements for the ventilators addressed electromagnetic compatibility, biocompatibility, risk mitigation (including safety), and sterilization [13].

In response to this call, five undergraduate students and multiple faculty members formed a team from the Faculties of Health Sciences and Engineering and Applied Science at Ontario Tech University. The team used daily video meetings and file sharing to complete a design based largely on readily available components with minimal assistance from additive manufacturing. When designing and building this mechanical ventilator, the goal was to keep the technical design simple and easy to manufacture and assemble. Additionally, we aimed to ensure ventilator operation was safe and user-friendly while passing all preliminary testing.

The mechanical ventilator was developed to deliver volume control ventilation (i.e., Continuous Mandatory Ventilation) at both hospital and remote emergency treatment settings (e.g., field hospital). It was designed to minimize resource consumption by operating with compressed air and oxygen while relying on a 12-volt (V) power source, such that it can operate on a standard car battery. The mechanical ventilator was equipped with a simple user interface, consisting of readily available liquid crystal display (LCD) screens. The screen displayed ventilator variables, allowing users to monitor patient vital signs and values, and set parameters such as respiratory rate, tidal volume, inspiratory pressures, alarms, etc. The four directional (up/down/left/right) and one “select” button allowed visualization of the patient’s status while facilitating ventilator parameter changes. Additional audio alarms were available to notify the user of a blockage or parameters out of range. These features allowed the ventilator to operate with minimal training for a variety of situations.

Inputs

Components and Materials

The necessary components to construct the mechanical ventilator may vary based on the location of where the ventilator is manufactured and the local availability of standard industrial automation and electrical components. This section will outline the components used in one of the prototypes built, the teams’ skills, and communication methods used to construct the prototype of the mechanical ventilator (Table 1).

**Table 1:** Pneumatic components. CFM: cubic feet per minute; DC: direct current; NPTF: national pipe taper fuel; V: Volts. Note: The sizes of the pneumatic ports and reservoirs were the minimum required for safe operation. Larger sizes could further improve the stability of the system.

Item/Component	Qty	Manufacturer Part #	Manufacturer	Website
¼” NPTF to ¼” Hose, Pneumatic Fitting	10	8819575	Princess Auto	https://bit.ly/2WxqyjH
¼” Pneumatic Hose Tee	4	8819518	Princess Auto	https://bit.ly/3gJLt9S
¼” Pneumatic Hose	1	8134868	Princess Auto	https://bit.ly/3zsJL42
18.5 CFM 12V DC Pneumatic Solenoid Valve	5	8192270	Princess Auto	https://bit.ly/3gMM0Ij
¼” Flow Control	3	8819492	Princess Auto	https://bit.ly/3DBGBxA

The electrical components required to assemble the mechanical ventilator are presented in Table 2.

**Table 2:** Electrical components. kPa: kilopascal; LCD: liquid crystal display.

Item/Component	Qty	Manufacturer Part #	Manufacturer	Website
16×2 LCD Display & Keypad Shield	1	16x2SHD-01	OSEPP	https://bit.ly/3EZ6Xdh
6V -180 deg Servo	3	900-00005	Parallax	https://bit.ly/3m5PA1M
Arduino Mega	1	MEGA 2560 REV3	Arduino	https://bit.ly/3i6R8Yn
Relay Module	5	WPM406	Velleman	https://bit.ly/3F6tSUn
±7kPa Differential Pressure Sensor	4	MPXV7007DP	NXP USA Inc.	https://bit.ly/2ZwJuAq
200kPa Gauge Pressure Sensor	2	ADP5151	Panasonic	https://bit.ly/2Zrfkhp
±100kPa Compound Pressure Sensor	1	ADP5100	Panasonic	https://bit.ly/2Zrfkhp

Infrastructure and Human Resources

The mechanical ventilator challenge required that every team be composed of both engineers and medical professionals. Each professional was responsible for a specific portion of the project. The engineering team (comprised of Dr. Sayyed Ali Hosseini, Dr. Ahmed Barari, and Benjamin DeBoer) was primarily responsible for the mechanical ventilator's design, build, and function. The medical team (comprised of respiratory therapists Marco Zaccagnini and Dr. Mika Nonoyama) was primarily responsible for providing their clinical experience regarding the design, functioning, and safety of using the ventilator in clinical practice. They also provided medical endorsement regarding the usability of the ventilator. Finally, the team recognized the need to test the mechanical ventilator in simulated scenarios for which Dr. Adam Dubrowski provided simulation expertise and equipment. 

The team built the mechanical ventilator during the COVID-19 pandemic, and all communication and consultation were done using Google Meets, a video-communication service developed by Google Inc [14], the primary online communication platform at Ontario Tech University.

Process

Design Process

The overall goal of the design cycles was to develop a design that effectively and efficiently addressed all necessary criteria of the open challenge, including manufacturing feasibility, low-cost, and rapid deployment time. The methodology included a series of closed-loops activities deployed within very short timelines for both the project management and technical details [15]. The initial alpha-prototype model was developed within multiple design loops, which was assessed for face validity by the medical members of the team. This was repeated twice, and a beta prototype was fabricated using the inputs described above.

Ventilator Pneumatic Design

**Figure 1:**
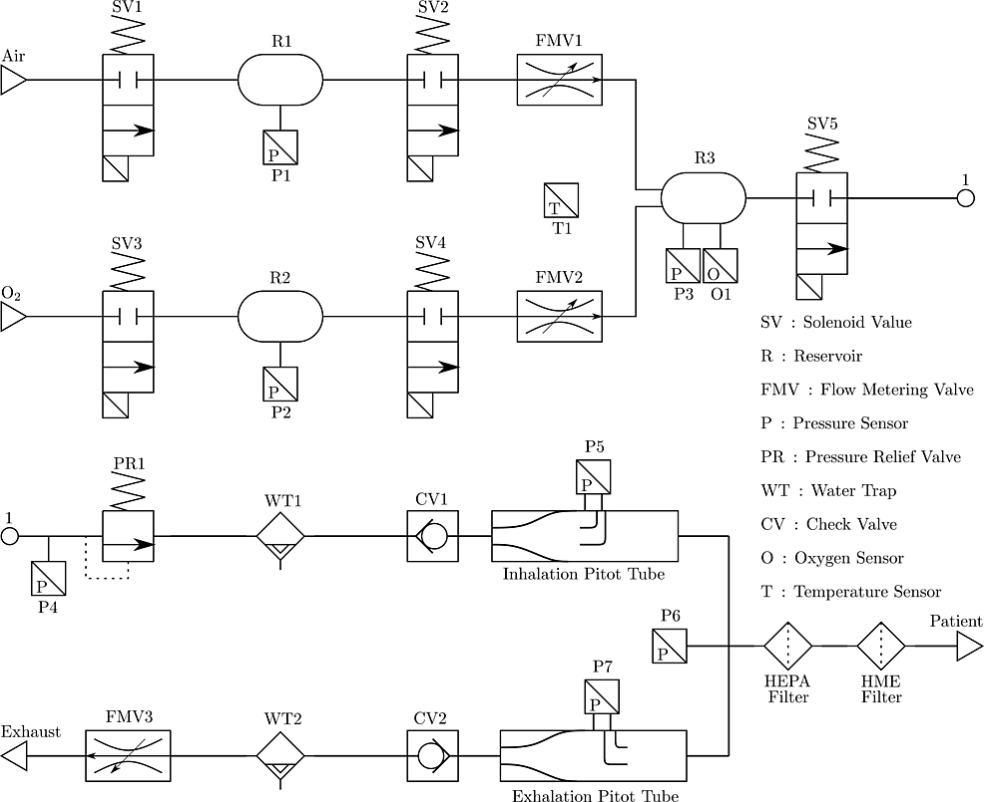
Prototype pneumatic diagram.

The mechanical ventilator was designed to be used with compressed air and oxygen (O_2_) using standard hospital pipeline supply or mobile tanks. The first step of the ventilator is to regulate the input gases into lower-pressure reservoirs (R1 and R2 of Figure 1) to 1-pound per square inch (PSI). The pressure regulation is conducted by selective solenoid activation of SV1 and SV3 until the required pressure was achieved. The final reservoir (R3) is then pressurized with the required fractional concentration of inspired oxygen (FiO_2_) mix by selective activation of SV2 and SV4 and verified by a single oxygen sensor (O1). The FiO_2_ level is controlled by the flow metering valves (FMV1 and FMV2) located between R1, R2 and R3. The final FiO_2_ mixture is then presented to the patient by activating the solenoid SV5, in which the mixture is subjected to a water trap and the required filters used in ventilation. When the process of inhalation is complete, the ventilator applies the required backpressure for exhalation. The back pressure is generated by metering the flow of the exhalation conducted by FMV3.

**Figure 2:**
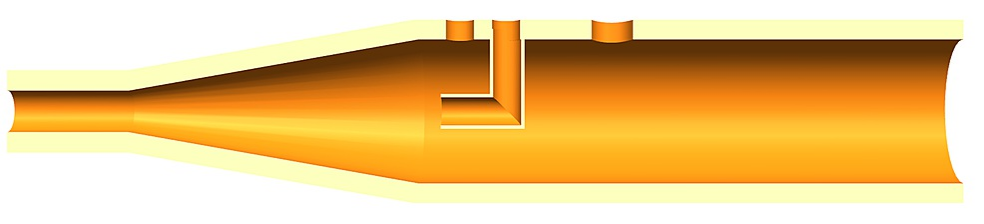
Inhalation Pitot tube design.

To control ventilation, health care professionals require various parameters and analysis metrics, such as inspiratory and expiratory pressure, tidal volume, FiO_2_ levels, and many more. These variables were measured using pressure sensors throughout the pneumatic circuit (P1-P7, and O1). It is common to use flow sensors to measure the tidal volume; however, they were not as accessible as pressure sensors at the time. To further reduce supply bottlenecks, tidal volume was calculated using a differential pressure reading from pitot tubes (Figure 2) on the pneumatic circuit’s inhalation and exhalation lines so health care professionals can monitor the values. The differential pressure sensors measure the pressure differential between the dynamic and static fluid pressure, allowing the determination of flow rate based on rudimentary fluid mechanic formulations. The formula is dependant on the density of the FiO_2_ mixture which can be determined by measuring the temperature (T1) of oxygen level (O1) of the gas in R3. The pitot tubes were the only part of the ventilator design that would require complex manufacturing methods, such as injection molding and additive manufacturing. Further, check valves, water traps, and pressure relief valves were added to the pneumatic circuit for redundancy and safety, see Figure 1 for the complete pneumatic diagram.

Ventilator Control System Design

**Figure 3:**
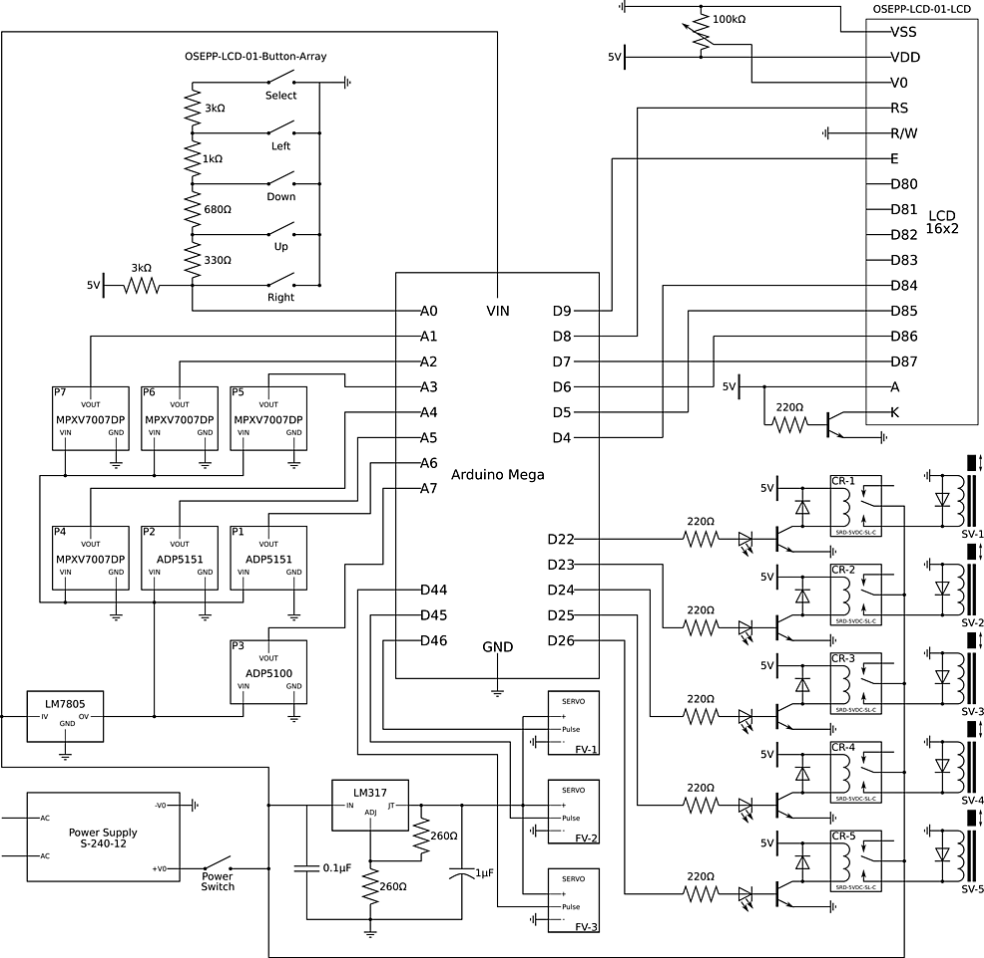
Ventilator electrical design.

A simple microcontroller programmed using the open-source Arduino software was used to control the mechanical ventilator process described above. The Arduino software inherently contains many software libraries and hardware drivers, making programming various microcontrollers simple. In the electrical design (see Figure 3), seven pressure sensors relayed data to the controller. Based on the parameters set by the user (e.g., respiratory therapist), five relays and three servos were activated to control five different solenoids and metering valves, respectively. The software was separated into an eight-step process, repeated continuously: closure of the exhalation port, pressure drop identification (representing the patients start of inhalation), the opening of the inhalation valve, set inspiratory tidal volume reached, closure of the inhalation valve, the opening of the exhalation valve, exhalation time complete, and ensuring exhalation complete by measuring the pressure. The controller also regulated the pressure in the R1 and R2 reservoirs, creates the FiO_2_ mixture in the R3 reservoir by reading the oxygen concentration from O1 and adjusting the FMV1 and FMV2, ensured adequate flow for inhalation, and created back pressure for exhalation throughout this process. Inhalation breaths are triggered when pressure sensor P6 detected a change in pressure below the level set by the user. A solenoid valve between the R3 reservoir and the test subject (SV5) was then activated until a set tidal volume was achieved. When the set tidal volume was reached, the inhalation valve closed, and the FiO_2_ mixing process was activated once more. Upon complete inhalation, the exhalation servo metering valve was also activated, allowing the simulated lung to exhale to the atmosphere while controlling the exhalation time and pressure.

**Figure 4:**
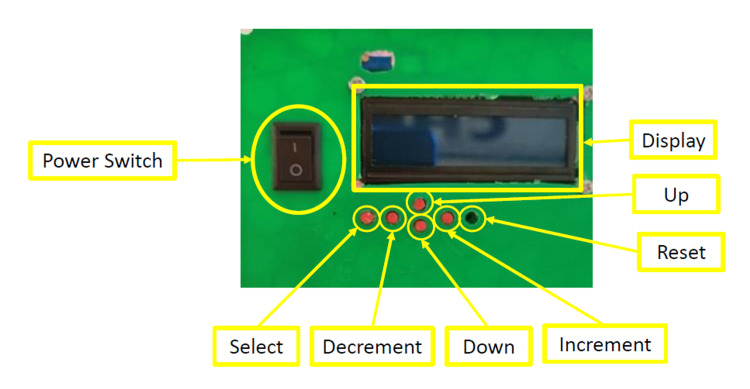
Mechanical Ventilator interface. The reset button is recessed to minimize false reset.

A simple interface was created to communicate with the end-user. The display and keypad (Figure 4) allowed the user to alter the set ventilation parameters and monitor the sensor reading throughout the device. The controllable parameters are inspired and expired pressure, inspiratory rate, and the FiO_2_ mixture. Each parameter had a minimum and maximum value the interface could not surpass to ensure patient safety. 

Ventilator Mechanical Design

**Figure 5:**
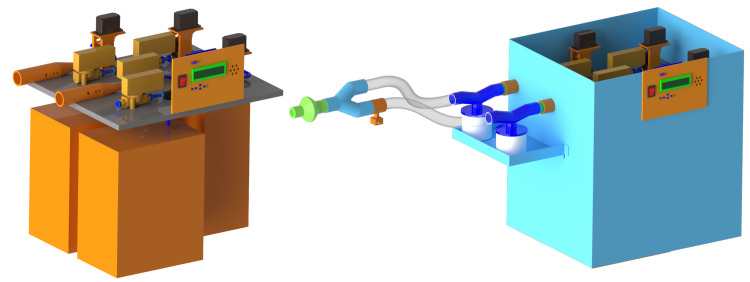
Full mechanical ventilator computer-aided design (CAD). Left: Tank and valve organization. Right: Final ventilator design.

The final ventilator design is shown in Figure 5, where the electrical and pneumatic systems were assembled on a single mounting plate before seating it within an enclosure. The mounting plate and enclosure material was aluminum, but other readily accessible materials can be used (e.g., plywood). An easy-to-follow assembly instruction manual was prepared so anyone with a basic knowledge of engineering could assemble the product. 

End-products/outcomes

Because of the time constraints imposed by the Montreal General Hospital Foundation and the Research Institute of the McGill University Health Centre challenge, the lack of access to proper testing facilities during the first COVID-19 lockdown and the time constraint of the competition, the team was forced to conduct an abridged equipment test. The testing that was completed included the user interface, pressure regulation, and eight-step operation triggers.

The user interface (Figure 4) was a vital part of a mechanical ventilator as this is how the user set various ventilator parameters. It was tested by setting various parameters (e.g., tidal volume, FiO_2_) using the up/down/left/right and select buttons. The user was successful at setting the desired parameters on the ventilator and was restricted when trying to pass the upper and lower safety limits set in the software. Once all the parameters were set, the interface continuously scrolled through the set parameters and measured sensor readings for the user to monitor.

We used a pressure source (consisting of a bicycle pump attached to a small external reservoir) to test the feasibility of pressure regulation using solenoid control. The pressure source was attached to both the oxygen and compressed air input of the ventilator. Two solenoid valves regulated the pressure in the R1 and R2 reservoirs to the required pressure via timed solenoid activation. The pressure readings within these reservoirs were monitored by differential pressure sensors between the reservoirs and the atmosphere and were displayed on the interface. The third reservoir was then pressurized with the correct FiO_2 _mixture using servo metering valves in series with solenoids valves until the required pressure (< 40 cm H_2_O) was achieved. The servo metering valves controlled the FiO_2_ mixture, where the solenoid valves controlled the pressure in the reservoir. Testing the system resulted in stable and accurate regulation of the three reservoirs. However, due to the COVID-19 restrictions, we could not validate the mixture of FiO_2 _because we didn’t have access to medical-grade O_2_ and O_2_ analyzers.

To enable the eight-step operation of the mechanical ventilator (described above in Ventilator Control System Design), a test subject (an undergraduate student working on the ventilator) placed the inspiratory line in their mouth (ensuring clean technique) and applied a small negative pressure. The system started applying the set inspiratory pressure through the inspiratory line until the set tidal volume was achieved. Once inhalation was completed, the exhalation metering valve activated and ensured adequate exhalation pressure, simulated by the test subject exhaling into the expiratory line. The process was repeated continuously without issue.

Before the mechanical ventilator is ready for patient use, further testing and validation are required to meet medical standards. Specifically, the ventilator requires testing to validate the control system’s operation and hardware components. These tests include validation of the oxygen/air mixture accuracy, flow readings, the system calculated tidal volume, and system regulated positive end-expiratory pressure (PEEP) using external measurement devices, along with a lifecycle analysis of the system.

## Discussion

This technical report described the design, development, and initial testing of a low-cost, prototype mechanical ventilator using basic, off-the-shelf components. Our current mechanical ventilator design is not intended to replace existing full functioning conventional mechanical ventilators but to offer an alternative option where a rapid influx of mechanical ventilators is required. Our team consisted of engineers, front-line clinicians, researchers, and undergraduate students who built the prototype mechanical ventilator for approximately $900 CAD (2020, covering the components). We chose to build this mechanical ventilator using off-the-shelf components to enhance the manufacturing speed. The industrial automation industry relies heavily on pneumatics for the daily operation of various machines. Therefore, to reduce the downtime in the manufacturing process, manufacturers and suppliers often have multiple backup pneumatic components readily available. Additional manufacturing, be it additive, formative, or subtractive, increases production time resulting in reduced ventilator production. Off-the-shelf components are vital for a quick and effective response in manufacturing. Thus, our mechanical ventilator, a controlled pneumatic device, was designed to include industrial automation solenoids and fittings to allow rapid production in an emergency, especially if resources like additive manufacturing or similar technologies are not available.

As it stands, the mechanical ventilator adheres to the tenants of effective concept design. An effective concept makes the goal of the product explicit and serves as the foundation upon which the product can be built in the future. Developing a concept demands a clear understanding of the type of problem to be solved, the ideal aesthetic style, the target audience, and the client’s needs (if working on an external project). In this report, we presented a concept design solution to a problem that was unexpected (i.e. shortages of ventilators due to the COVID-19 pandemic). The initial problem and design parameters were very well articulated by the Montreal General Hospital Foundation and the Research Institute of the McGill University Health Centre challenge, and the work presented here adhered to the challenge. We were not constrained by the aesthetics of the design, as this was not an important part of the challenge. The target audience and client's needs were also well articulated by the challenge and addressed at the team level by the diversity of the team. That is, in addition to undergraduate students, our team consisted of clinicians, engineers and other health professionals and researchers to address all aspects of the design. Because the intention was not to develop a fully functional and/or commercial level prototype (for example Technology Readiness Scales 7 or greater), and because of the lack of proper simulated and clinical testing, our ventilator should be considered a concept design and, in the future, it may serve for our or other teams to carry additional development.

Future research on our (or any rapidly developed mechanical ventilator) should include testing human factors/ergonomics related to operating the ventilators to minimize errors related to design choices. Individuals involved in researching and developing mechanical ventilators should consider aspects related to the user, the environment, the tasks and the risks when conducting clinical assessments to test the overall performance and usability of the mechanical ventilator [16,17]. These assessments could be based on established global and comprehensive performance evaluation framework models and checklists developed for ICU mechanical ventilators and/or guidance provided by the Charted Institute of Ergonomics & Human Factors group on formative usability testing of rapidly developed mechanical ventilators [16-20]. Technical performances could be completed with a lung simulator along with a respiratory monitor for parameter measurements. Simulation tests could be repeated under normal, restrictive, and obstructive lung conditions, each with specific pass criteria. Usability and perceived workload could be assessed by health care provider end-users (e.g., respiratory therapists) by performing timed tasks related to the monitoring and settings of the ventilators. Measures such as the 10-item System Usability Scale (SUS) [21] and the NASA-TLX [22] have been used in the past for similar assessments. Human factors/ergonomics testing should be conducted with the end-user (e.g., physician, respiratory therapist) to minimize problems such as accidental disconnections, minimize the potential to miss critical alarms, minimize musculoskeletal health risk to stakes (e.g., screens placed awkwardly) or misreading information on the screen. This type of information could help the engineering team integrate modifications for future iterations of the rapid mechanical ventilator designs.

The virtual collaborative process of creating this mechanical ventilator provided lessons learned in the context of online learning. The spread of COVID-19 has led to unprecedented measures and restrictions by governments, businesses, and research institutions to limit the spread of the COVID-19 virus. In addition to restricting travel and cancelling large events, a growing number of industries are now rethinking their ways of working. While certain sectors, such as medical staff, are still working on-site, most of the remaining workforce (e.g., researchers) are encouraged or even mandated to work remotely. Working remotely under these circumstances means adapting to a new environment, battling a new set of distractions, and experiencing an unprecedented fusion of work and private life. We found that working under these circumstances was possible, and the efficiencies were related to frequent team meetings at three levels: entire team meetings weekly with structured presentations, daily sub-team meetings, and daily project leadership meetings. This technical report can serve as a blueprint to determine the most feasible way to construct a mechanical ventilator for other possible pandemic or mass casualty scenarios.

## Conclusions

Our team successfully designed, built, and conducted preliminary testing on a low-cost, prototype mechanical ventilator using basic, off-the-shelf components within 10 days. While there is no current need to use these low-cost ventilators on patients, it is important to document the blueprints, design processes and available outcomes to improve future iterations and add to the growing body of open-source evidence.

The ventilator prototype was built using parts that could be ordered online through local vendors or international retailers and received within days, minimizing the dependence on manufacturing methods. Overall, developing a mechanical ventilator based on the off-the-shelf components is an important strategy for rapid development. Various industries have mass-produced industrial and consumer products that can be used in emergency medical environments. As engineers and researchers, we must exploit all the readily available tools and products to achieve a quick, robust, and life-changing product within a matter of days.
